# Supplementary material for: bamSliceR: a Bioconductor package for rapid, cross-cohort variant and allelic bias analysis
Source: Bioinform Adv. 2025 Apr 28;5(1):vbaf098. doi: 10.1093/bioadv/vbaf098 (PMC12089696; doi:10.1093/bioadv/vbaf098)
Supplement: vbaf098_Supplementary_Data [file vbaf098_supplementary_data.docx]

**Supplemental Data**

# Table S1. Co-occurrence status of *H3K27M* and *IDH2* mutations in TARGET-AML and BEAT-AML.

Identification of 9 pAML patients that harbored K27M mutation on Histone 3 genes based on evidence of VAF > 0.15 and total read depth > 8 and whether the mutation can be captured by both RNA-seq and WGS data. Co-occurrence of *H3F3A* (H3.3) *K27M* and *IDH2 R172K* are shown in 2 pAML patients. Co-occurrence of *H3K27M* (H3.3 & H3.1) and *IDH2 R140Q/R172K* are shown in 5 adult AML patients.

|  |  | **H3K27M** | | | | | **IDH2** | | | | |
| --- | --- | --- | --- | --- | --- | --- | --- | --- | --- | --- | --- |
|  | **CASE_ID** | **SYMBOL** | **DNA-vaf-T1** | **DNA-vaf-T2** | **RNA-vaf-T1** | **RNA-vaf-T2** | **SYMBOL** | **DNA-vaf-T1** | **DNA-vaf-T2** | **RNA-vaf-T1** | **RNA-vaf-T2** |
| **H3.3** | PARBTV | H3F3A | 47% | 48% | 47%% | 47% | R172K | 38% | 48% | 56% | 59%% |
|  | PAVDMY | H3F3A |  |  | 49% | 47%/47% | R172K |  |  | 39% | 40%/48% |
| **H3.1** | PAPVGE | HIST1H3C |  |  | 58%% |  | - |  |  | - |  |
|  | PAUZTH | HIST1H3J^K27I^ |  |  | 0% | 38%% | - |  |  | - | - |
|  | PAUUPR | HIST1H3I |  |  | 0% | 35%% | - |  |  | - | - |
|  | PAKWCU | HIST1H3D |  |  | 17%% |  | - |  |  | - |  |
|  | PAXFAG | HIST1H3C |  |  | 54%% |  | - |  |  | - |  |
|  | PAXKAL | HIST1H3I |  |  | 50% |  | - |  |  | - |  |
|  | PATFGK | HIST1H3C |  |  | 50% |  | - |  |  | - |  |

|  |  | **H3K27M** | | | | **IDH2** | | | |
| --- | --- | --- | --- | --- | --- | --- | --- | --- | --- |
|  | **CASE_ID** | **SYMBOL** | **DNA-vaf** | **RNA-vaf** | **TS-vaf** | **SYMBOL** | **DNA-vaf** | **RNA-vaf** | **TS-vaf** |
| **H3.3** | 2148 | H3F3A | 29% | 47% |  | R140Q | 40% | 52% |  |
| **H3.1** | 2354 | HIST1H3C | 34%/33% | low-exp |  | R172K | 36%/31% | 47% |  |
|  | 2429 | HIST1H3B | 33%/47% | 35.29%/100% |  | - | - | - |  |
|  | 2498 | HIST1H3C | 37% | 38% |  | R140Q | 48% | 47% |  |
|  | 2530 | HIST1H3B | 40% | low-exp |  | R172K | 46% | 49% |  |
|  | 2611 | HIST1H3D |  | 58% | 39% | - |  | - | - |
|  | 2721 | HIST1H3B |  |  | 36% | R172K |  |  | 36% |

## **Table S2** *H3K27* variants: published work (n=1049) and TARGET/BEAT-AML cohorts (n = 2934, via *bamSliceR*)

Lehnertz et al. *Blood* *2017* documented 2 adult AML patients. Boileau et al. *Nat Commun 2019* documented 4 adult AML patients. We documented 16 AML patients from TARGET and BEAT AML cohorts.

| **Cohort** | **Cohort Size (n)** | **WGS/WXS (n)** | **RNAseq (n)** | **DNA Methylation (n)** | **H3K27M/I (%/n)** |
| --- | --- | --- | --- | --- | --- |
| **Lehnertz et al. *Blood* (2017)** | | | | | |
| **Leucegene** | 415 |  | 415 |  | 0.48%/2 |
| **Boileau et al. *Nat Commun* (2019)** | | | | | |
| **Toronto** | 312 | 312 |  |  | 0.64%/2 |
| **Lebanon** | 122 | 122 |  |  | 0.8%/1 |
| **TCGA** | 200 | 200 | 200 | 200 | 0.5%/1 |
| **Identification of H3K27M in BEAT-AML and TARGET-AML** | | | | | |
| **TARGET 20/21** | 2045 | 365 | **2281** | 2000 | 0.4%/9 |
| **Beat-AML** | 826 | 798 | **653** |  | 0.8%/7 |

## Table S3

**Human *MLLT1* YEATS domain insertion/deletion variants identified in the pan-TARGET cohort.**

| **Chromosome** | **POS** | **SYMBOL** | **AAchange** | **REFCODON** | **VARCODON** | **REFAA** | **VARAA** | **alt_count** | **totla_count** | **VAF** | **patient_id** |
| --- | --- | --- | --- | --- | --- | --- | --- | --- | --- | --- | --- |
| **chr19** | 6230649 | MLLT1 | V114VNHL | GTG | GTGAACCACCTG | V | VNHL | 5 | 51 | 0.09804 | PASBGZ |
| **chr19** | 6230642 | MLLT1 | H116HLRP | CAC | CACCTGCGCCCC | H | HLRP | 2 | 460 | 0.00435 | PANGJY |
| **chr19** | 6230645 | MLLT1 | N115NPLR | AAC | AACCCCCTGCGC | N | NPLR | 4 | 470 | 0.00851 | PANGJY |
| **chr19** | 6230645 | MLLT1 | N115NHLR | AAC | AACCACCTGCGC | N | NHLR | 55 | 470 | 0.11702 | PANGJY |
| **chr19** | 6230640 | MLLT1 | HL116L | CACCTG | CTG | HL | L | 2 | 134 | 0.01493 | PALHVV |
| **chr19** | 6230646 | MLLT1 | N115NHLH | AAC | AACCACCTGCAC | N | NHLH | 2 | 76 | 0.02632 | PASBPK |
| **chr19** | 6230582 | MLLT1 | LL135L | CTCCTG | CTG | LL | L | 2 | 175 | 0.01143 | PAUMUZ |
| **chr19** | 6230611 | MLLT1 | NP126P | AACCCC | CCC | NP | P | 2 | 370 | 0.00541 | PAUHGM |
| **chr19** | 6230616 | MLLT1 | TFN123N | ACCTTCAAC | AAC | TFN | N | 2 | 48 | 0.04167 | PAWVPZ |
| **chr19** | 6230620 | MLLT1 | TF123F | ACCTTC | TTC | TF | F | 2 | 690 | 0.00290 | PAVDXR |
| **chr19** | 6230574 | MLLT1 | AG138G | GCCGGC | GGC | AG | G | 2 | 175 | 0.01143 | PABYYR |
| **chr19** | 6230570 | MLLT1 | GG139G | GGCGGG | GGG | GG | G | 2 | 1047 | 0.00191 | PAUWZR |

## **Figure S1**

**Figure S1. *bamSliceR* Data Visualization Functionality**

**A-D** Example of automatically generation of *Oncoplot, survival analysis, VAF distribution* and *Mutual Exclusivity analysis.* **B.** VAF plotting of different mutations of *IDH2* at either *R140* or *R172* ordering by median of VAF. *R140Q* and *R172K* are most prevalent and *R172K* mutation are always clonal which are usually have mean allele frequency around ~50% assuming pure sample (VAF plotting of *H3K27M* and *DNMT3A* are in Supplementary Figure S1 and S2 ). **C.** Kaplan meier curve by grouping samples based on mutation status (WT vs. DNMT3A) in both TARGET and BEAT AML cohorts (n = 2934). **D.** Mutual exclusivity plot by pair-wise Fisher’s Exact test detected *H3-3A K27M* and *IDH2 R172K* are co-occuring mutations (p < 0.0032).


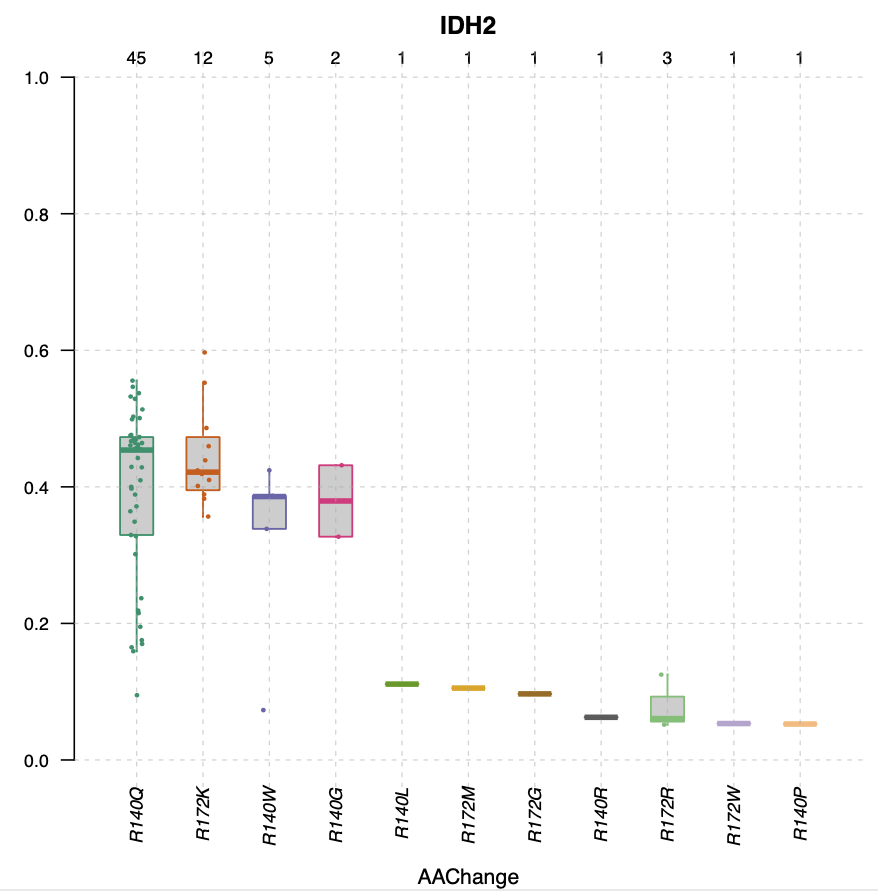

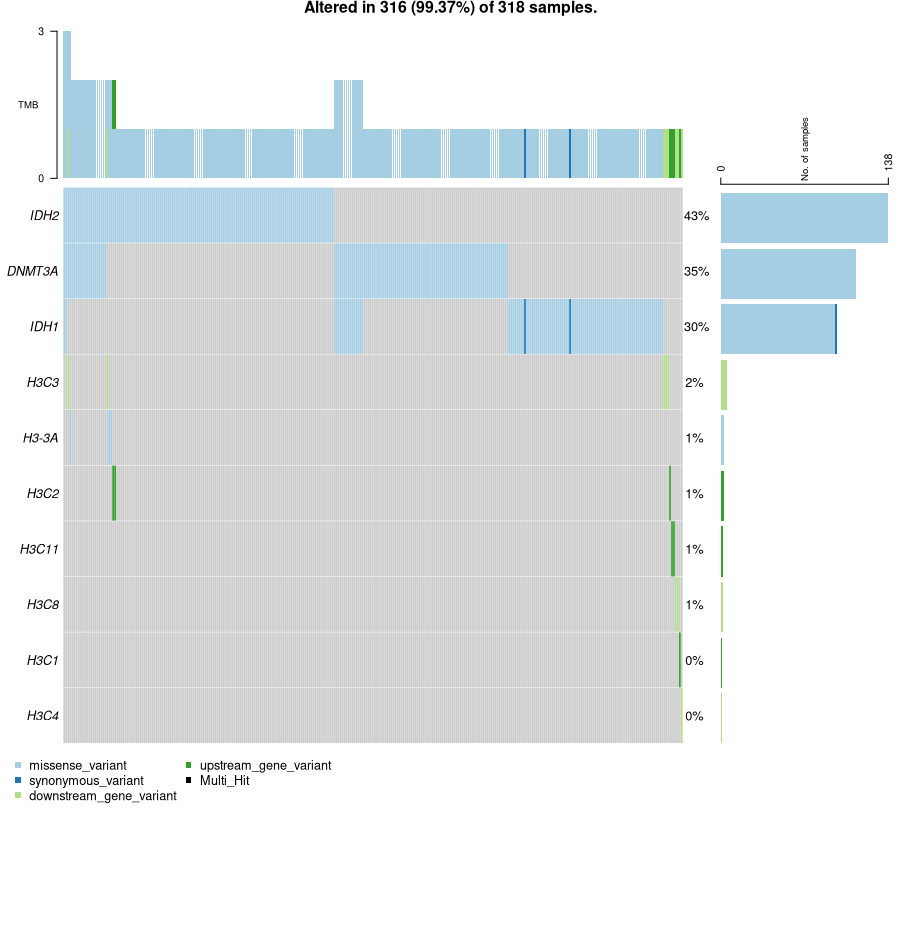

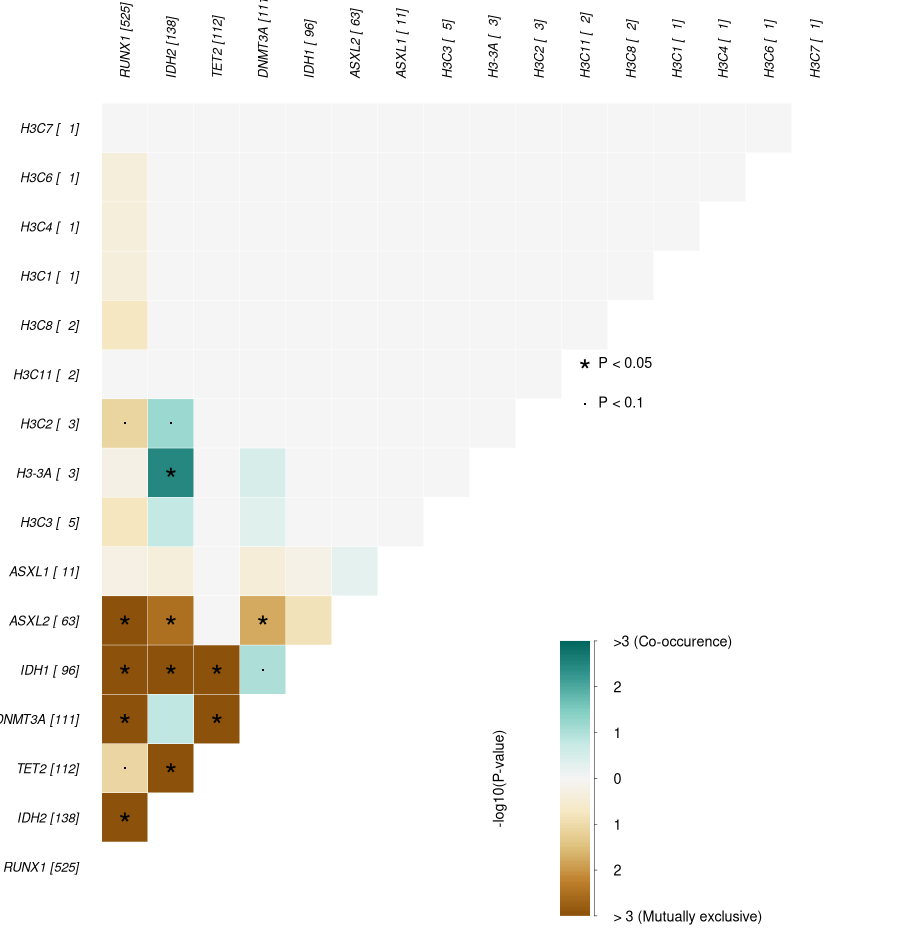

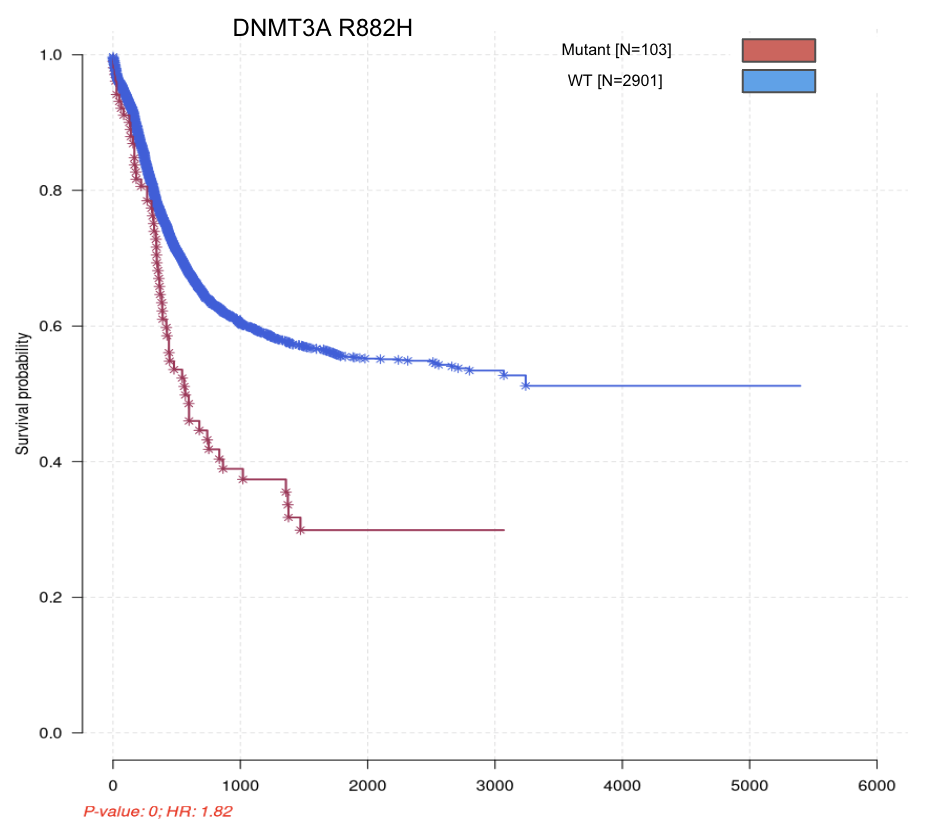


A.

B.

C.

D.

##

## Figure S2

**VAF distribution of DNMT3A variants.**

## Figure S2

**VAF distribution of DNMT3A variants.**

## Figure S2

# Figure S2

## **VAF distribution of DNMT3A variants.**

**
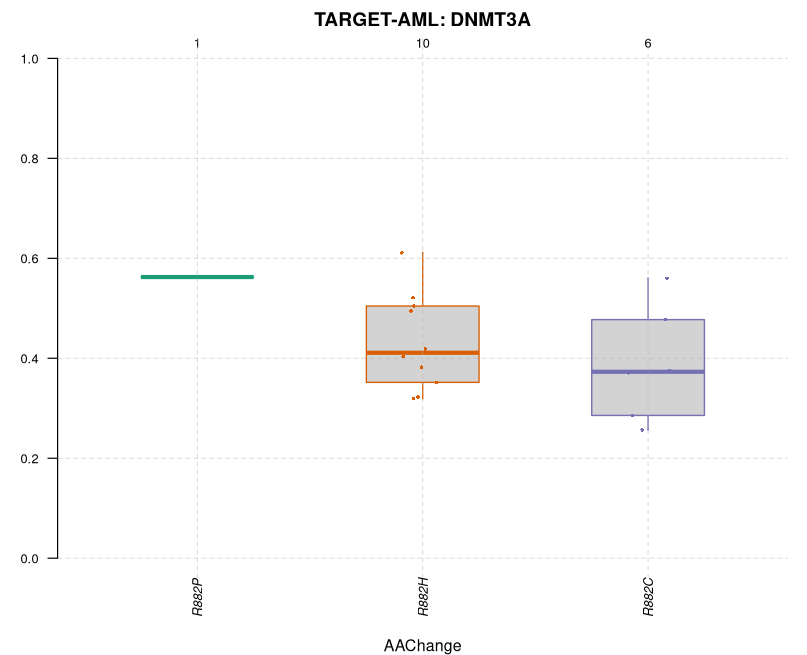
**

# Figure S3

## **VAF distribution of H3K27 variants.**

**
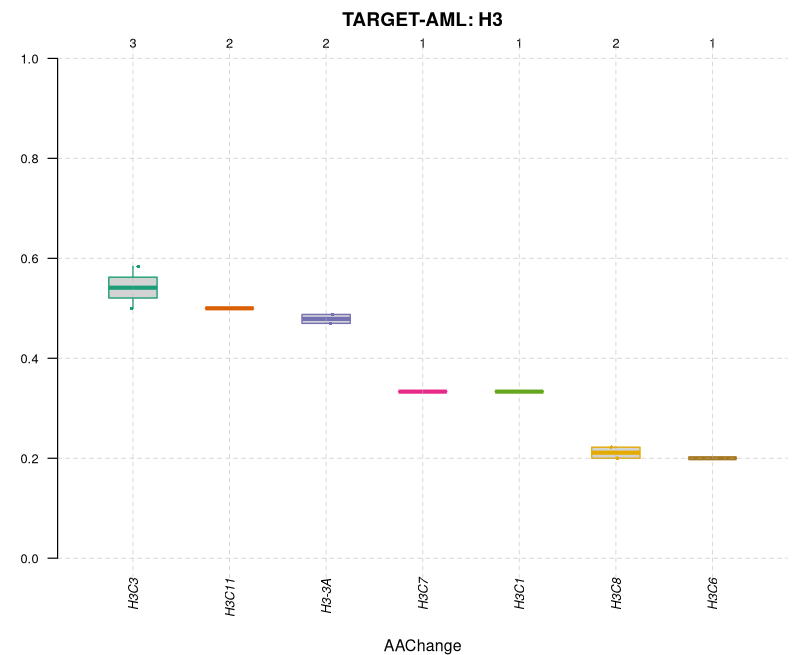
**

# Figure S4


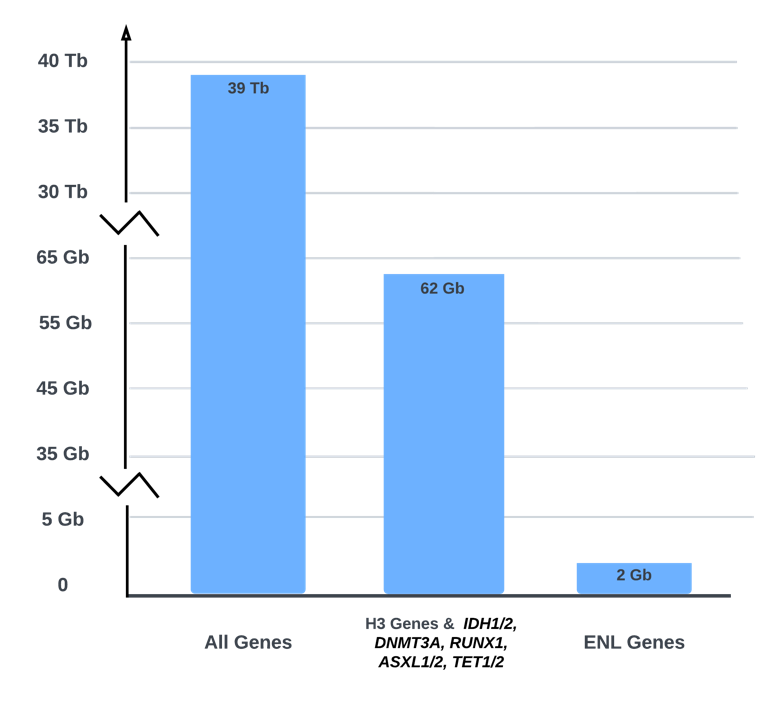


**Figure 5.** bamSliceR is designed to significantly reduce the space required for analyzing BAM files by slicing them at specified genomic regions.

#
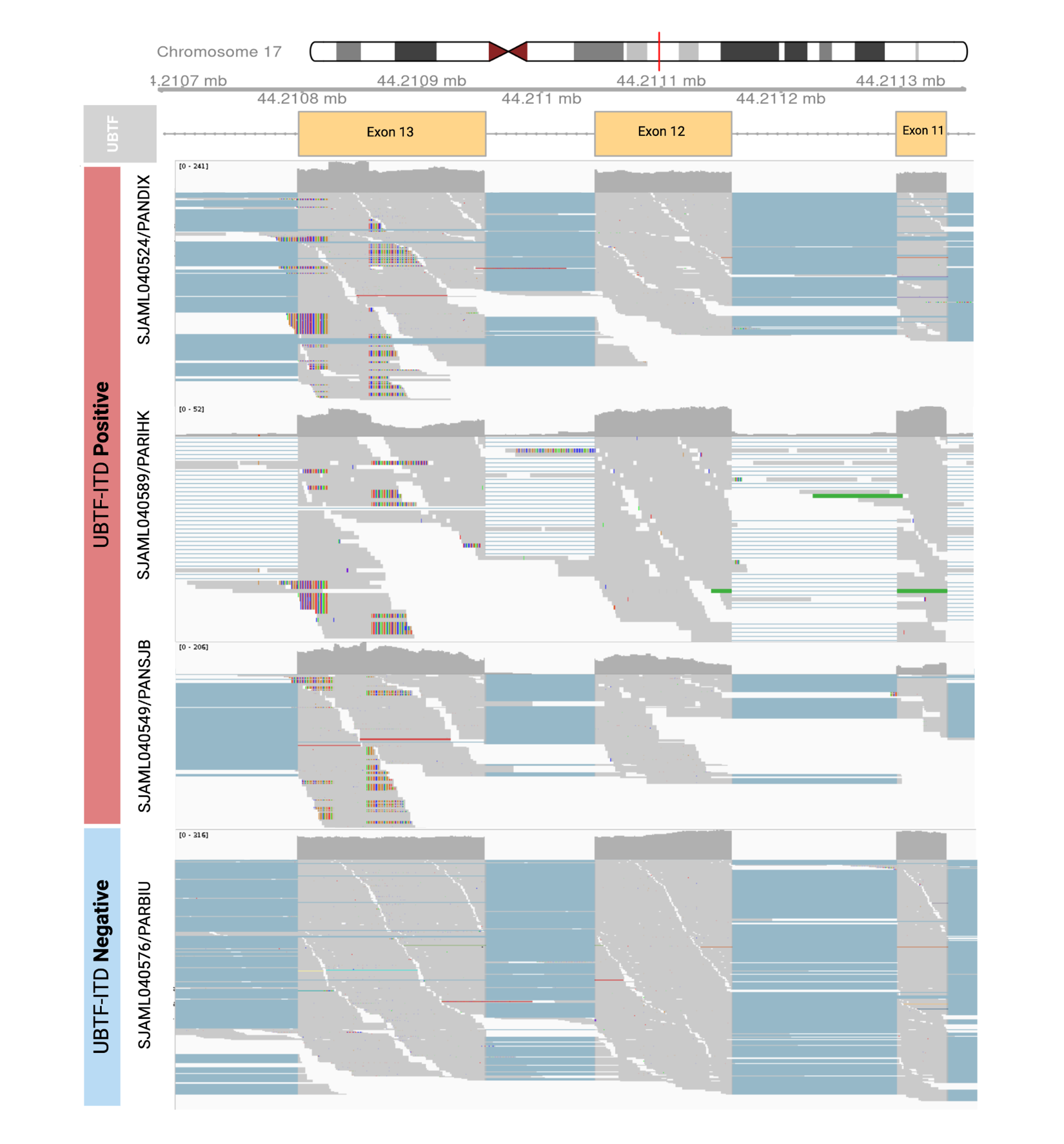
Figure S5

**Figure S4.** Positive and Negative controls of UBTF-ITD events confirmed in TARGET AML cases. The matched StJude and TARGET IDs of patients are presented. All samples are from primary AML bone marrow. The UBTF-ITD status is confirmed in Umeda, M. et al. (2022) using CICERO(v1.7.0), INDEL detection, and soft-clipped read counts ratio. Integrative Genomics Viewer (IGV) visualization showing soft-clipped reads and increased coverage in UBTF exon 13 in UBTF-ITD positive patients but not UBTF-ITD negative patients.


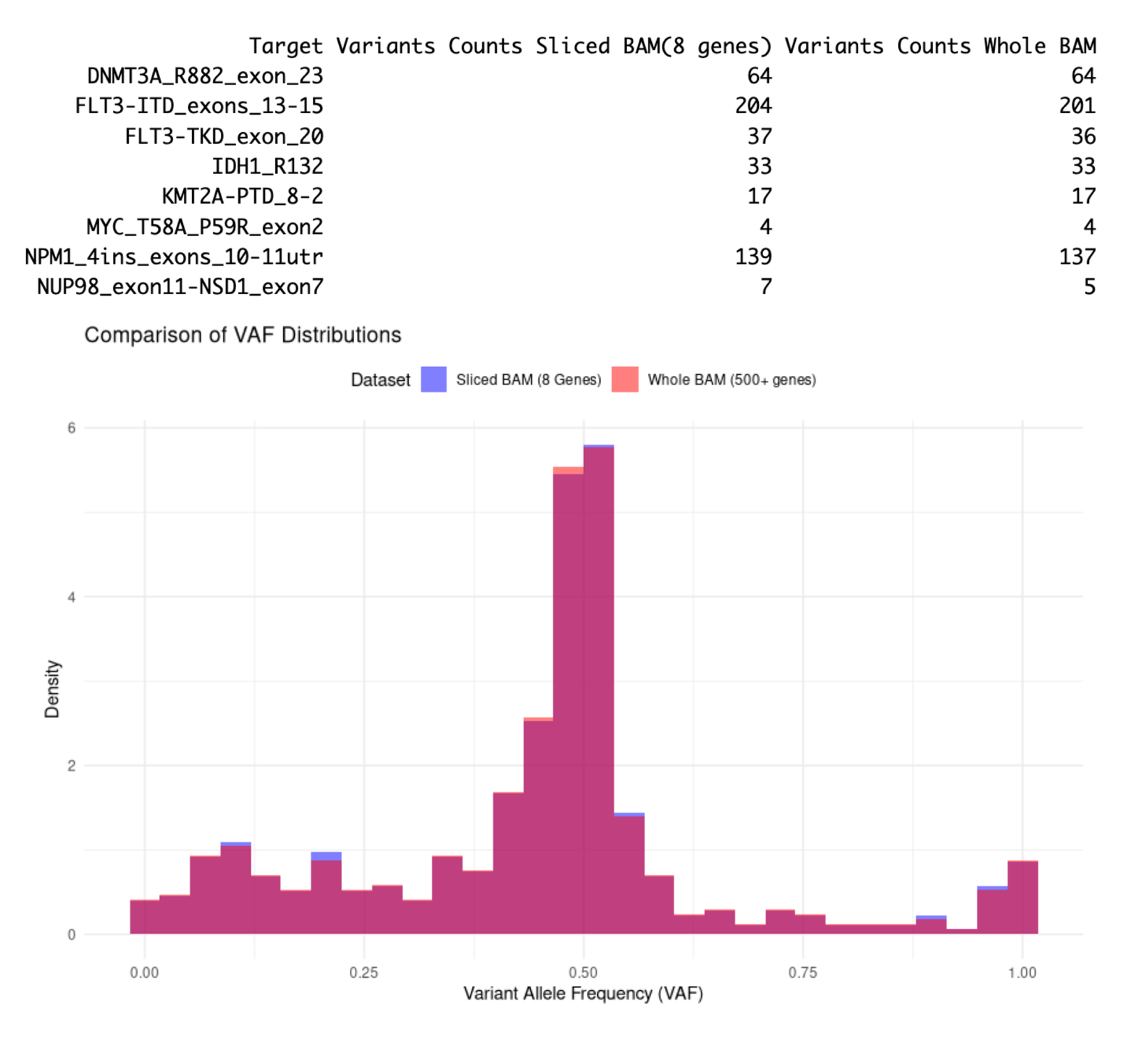


**Figure S5. Comparison of Variants Detection by Km using sliced BAM and Whole BAM (500+ genes) files.** BAM files (Leucegene RNA-seq, n = 452) contains reads aligned to 8 genes (*DNMT3a*, *FLT3*, *IDH1*, *KMT2A*, *MYC*, *NPM1*, *NUP98*, *NSD1*)  and 500+ genes are sliced from whole BAM files generated using aligner STAR. Km was run on 8 target variants using the sequencing provided by Km github repository (<https://github.com/iric-soft/km/tree/master/data/catalog/GRCh38>). The number of detected variants for each target and the VAF distribution are nearly identical by using sliced BAM files.


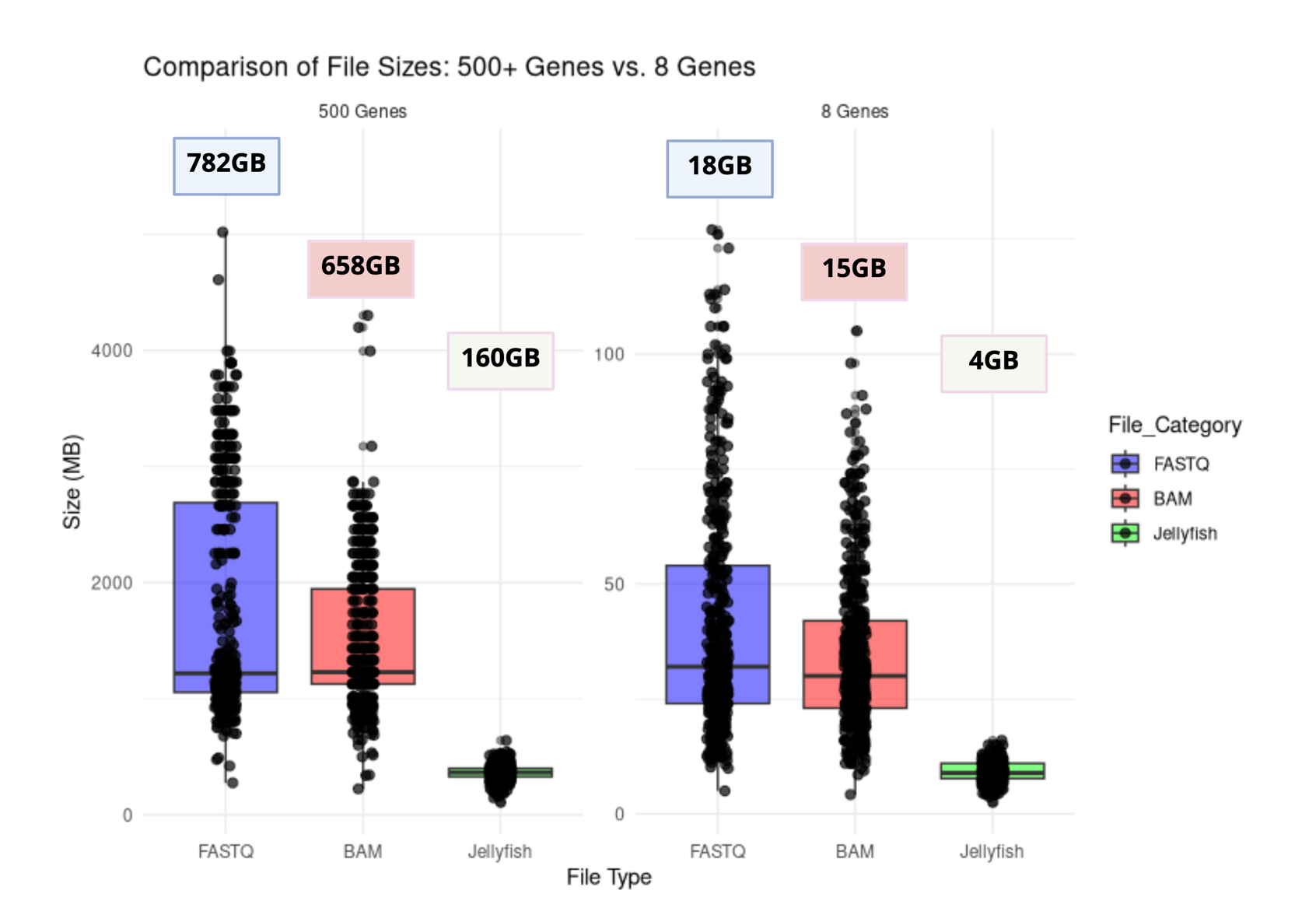


**Figure S6. Comparison of Files Size in different formats: FASTQ vs. BAM vs. K-mer Count Table.** K-mer count tables are created by Jellyfish, with k = 31 bp and minimum k-mer count of 2. BAM files produced using aligner STAR. Total size of files in each format can be drastically reduced when BAM files are ready on remote database and BAM slicing API is available.


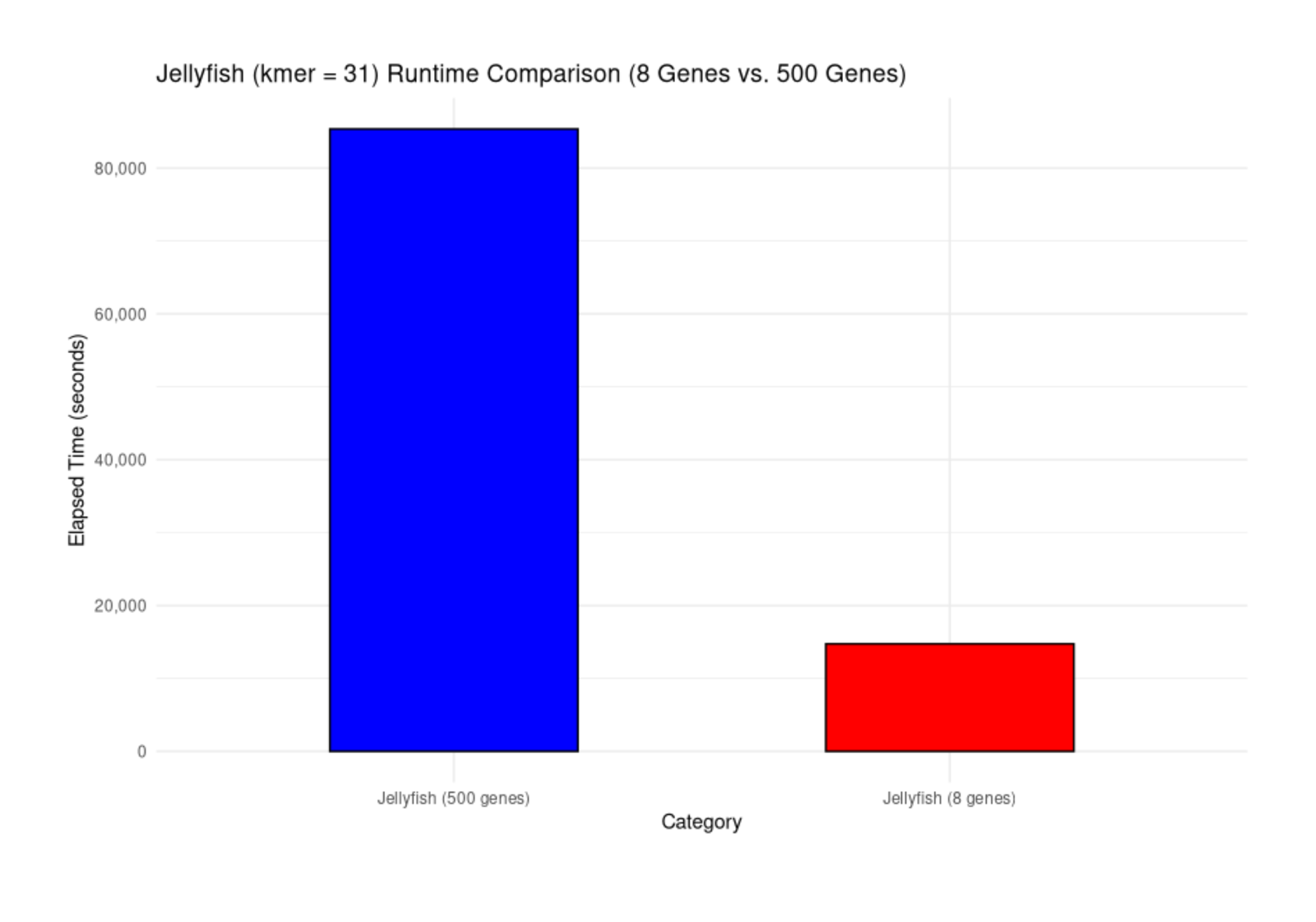


**Figure S7. Comparison of Runtime of precomputing count tables using sliced BAM and Whole BAM (500+ genes) files.** Km can call variants without alignment. Instead, Km requires precomputing K-mer count tables generated by Jellyfish using raw reads in FASTQ format. It required 23 h and 42 min to generate count tables from 782 GB FASTQ files. The runtime can be reduced to 4 h and 5 min by slicing 8 target genes if BAM files are already available which yields 18 GB FASTQ files.


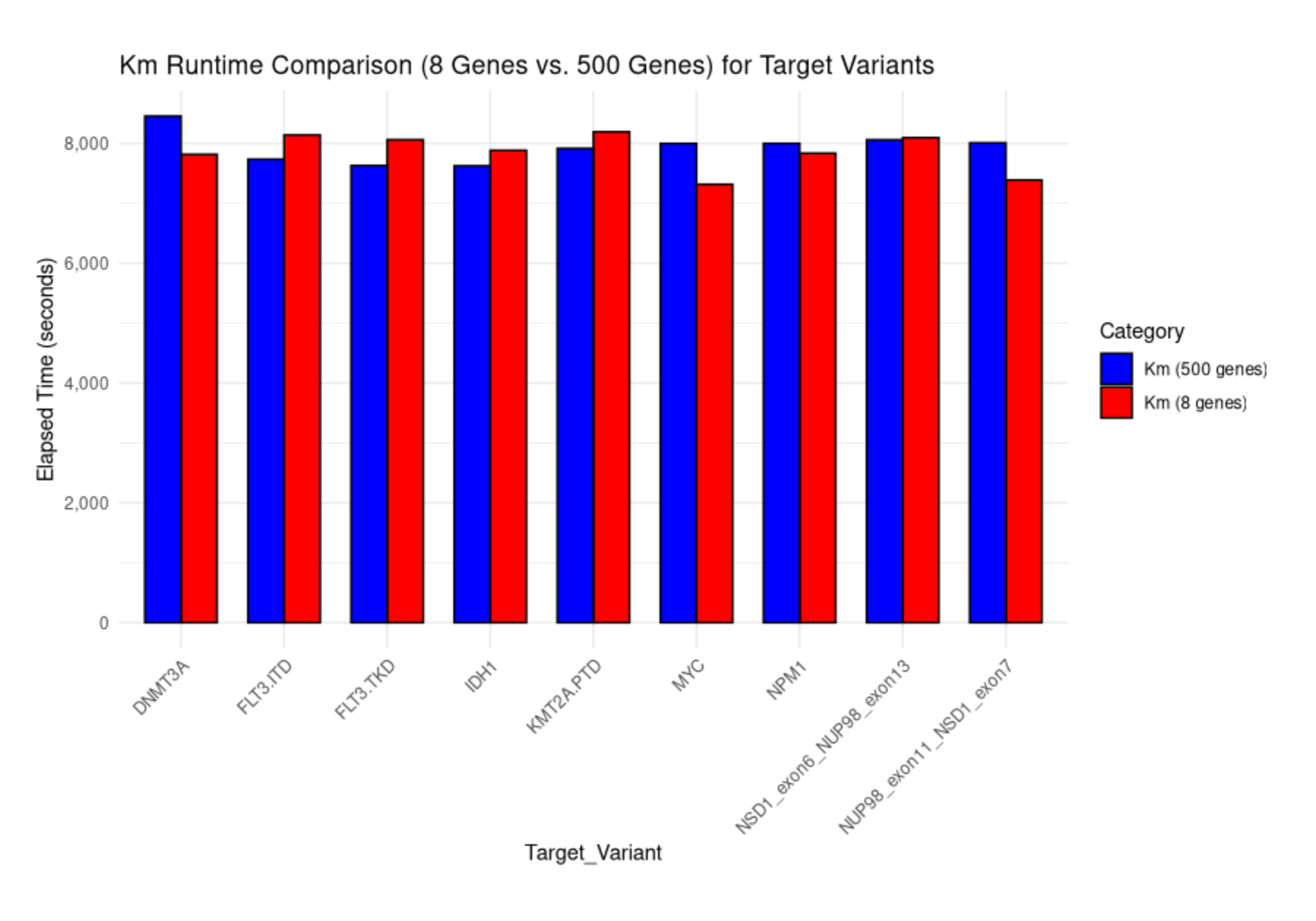


**Figure S8. Comparison of Runtime of variant detection step in Km using sliced BAM and Whole BAM (500+ genes) files.** The runtime of the second step of Km involves variant detection that does not benefit from BAM slicing.


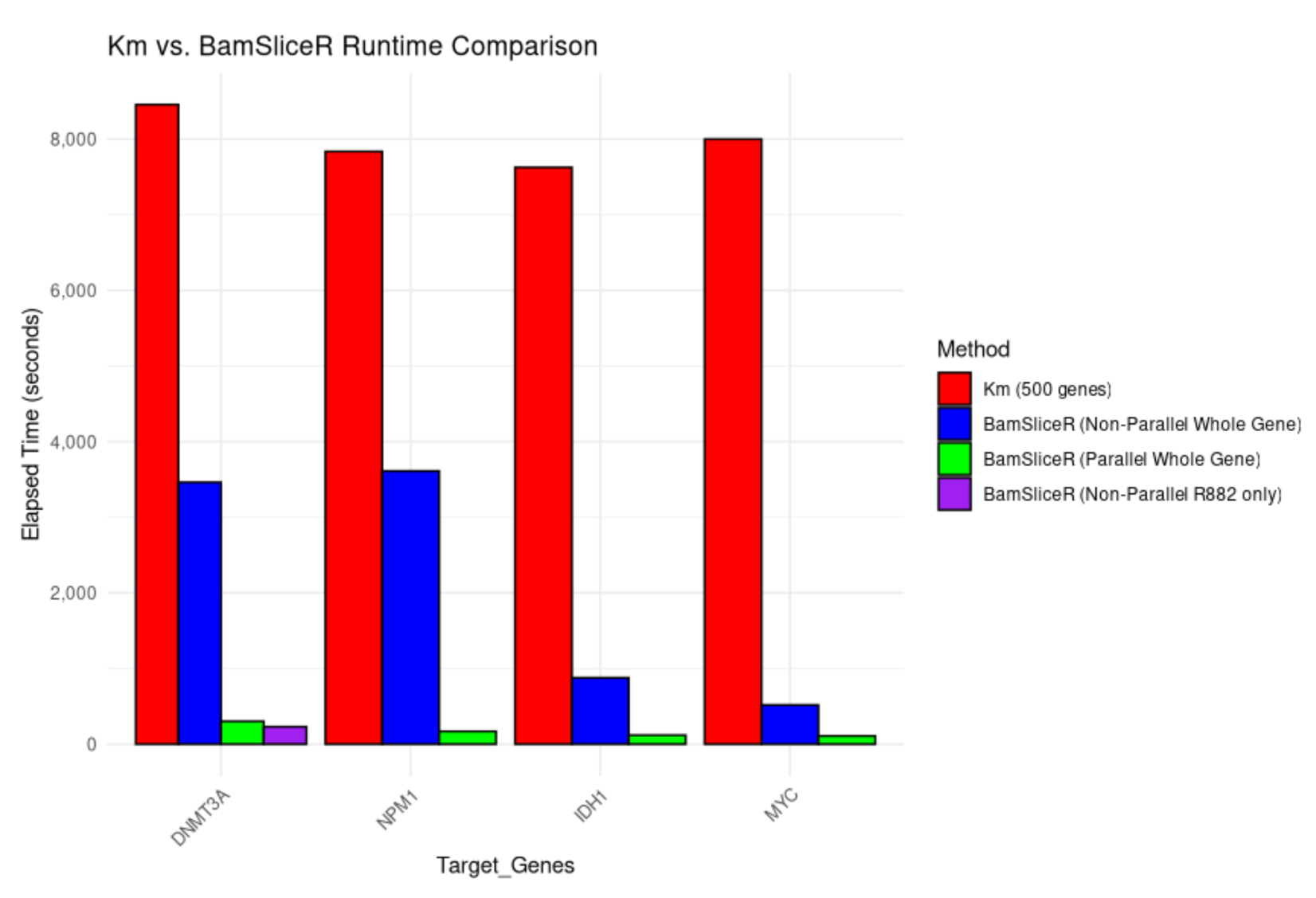


**Figure S9. Comparison of Runtime between Km and bamSliceR.** Km and bamSliceR are run on 452 Leucegene samples to detect SNP and small INDEL: *DNMT3A R882*, *IDH1 R132*, *MYC* and NPM1 *4-base insertion*. In general, the pileup-appaoch used by bamSliceR is faster than Km. And bamSliceR provide options of parallel computing on both BAM files and genomic ranges queried if multi-thread machines or HPC are available which can further speed up the process.

**
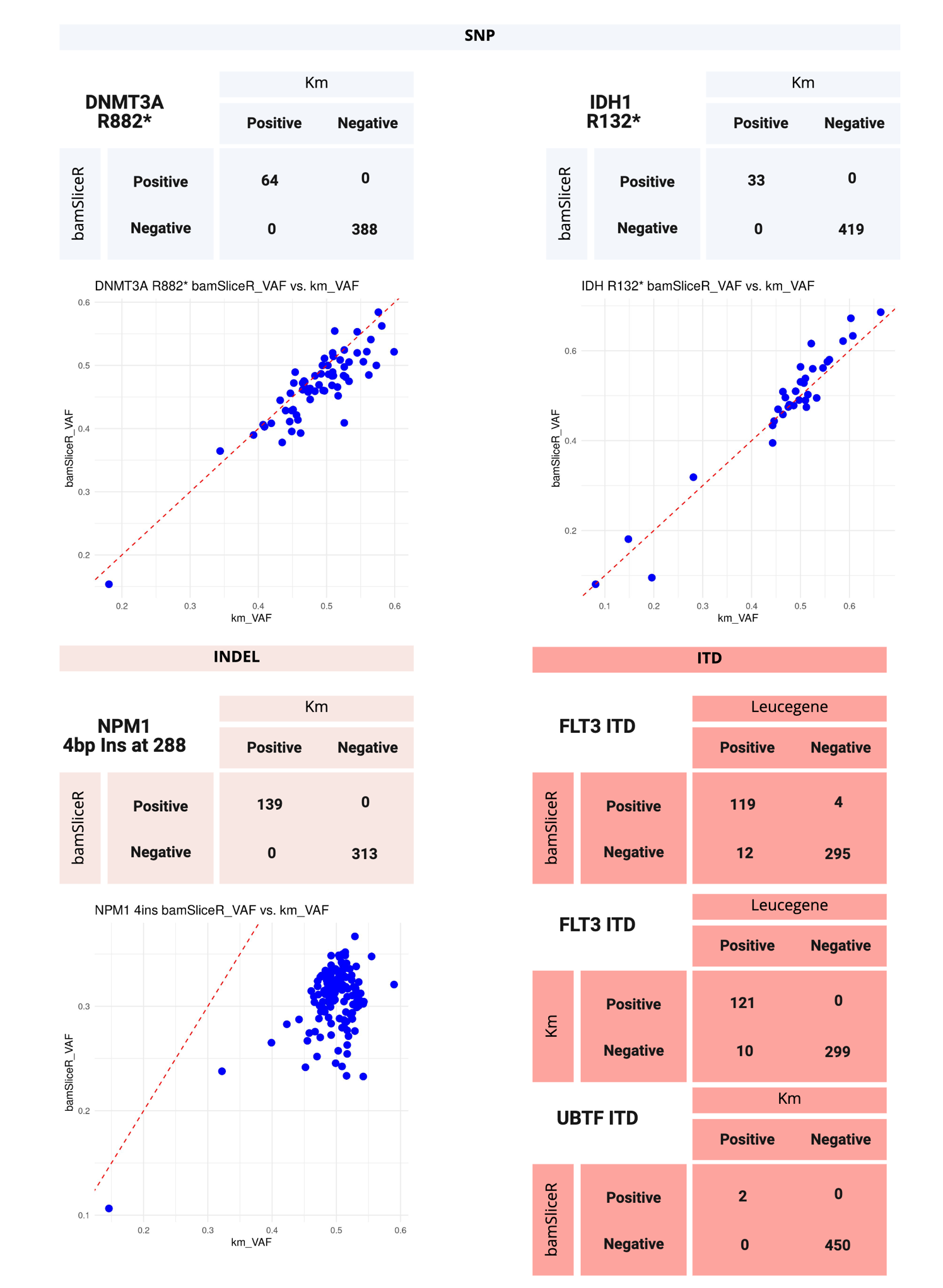
**

**Figure S10. Contingency tables Different type of Variants and VAF Comparison.** The number of SNP and small INDEL detected by Km and bamSliceR are the same. For NPM1 4pb Ins at 288 locations, bamSliceR estimated overall lower VAF of the variants. The FLT3-ITD variant have been experimentally validated by the Banque de Cellules Leucémique du Québec (BCLQ, <https://bclq.org/>). The much rarer variant UBTF-ITD has never been documented in the Leucegene cohort before.
